# Adenosine A1 Receptors Promote *Vasa Vasorum* Endothelial Cell Barrier Integrity via G_i_ and Akt-Dependent Actin Cytoskeleton Remodeling

**DOI:** 10.1371/journal.pone.0059733

**Published:** 2013-04-16

**Authors:** Nagavedi Siddaramappa Umapathy, Elzbieta Kaczmarek, Nooreen Fatteh, Nana Burns, Rudolf Lucas, Kurt R. Stenmark, Alexander D. Verin, Evgenia V. Gerasimovskaya

**Affiliations:** 1 Vascular Biology Center, Georgia Regents University, Augusta, Georgia, United States of America; 2 Division of Pulmonary Medicine, Georgia Regents University, Augusta, Georgia, United States of America; 3 Center for Vascular Biology Research, Department of Surgery, Beth Israel Deaconess Medical Center, Harvard Medical School, Boston, Massachusetts, United States of America; 4 Department of Pediatrics, University of Colorado Denver, Aurora, Colorado, United States of America; 5 Department of Pharmacology and Toxicology, Georgia Regents University, Augusta, Georgia, United States of America; University of Colorado Denver, United States of America

## Abstract

**Background:**

In a neonatal model of hypoxic pulmonary hypertension, a dramatic pulmonary artery adventitial thickening, accumulation of inflammatory cells in the adventitial compartment, and angiogenic expansion of the *vasa vasorum* microcirculatory network are observed. These pathophysiological responses suggest that rapidly proliferating vasa vasorum endothelial cells (VVEC) may exhibit increased permeability for circulating blood cells and macromolecules. However, the molecular mechanisms underlying these observations remain unexplored. Some reports implicated extracellular adenosine in the regulation of vascular permeability under hypoxic and inflammatory conditions. Thus, we aimed to determine the role of adenosine in barrier regulation of VVEC isolated from the pulmonary arteries of normoxic (VVEC-Co) or chronically hypoxic (VVEC-Hyp) neonatal calves.

**Principal Findings:**

We demonstrate via a transendothelial electrical resistance measurement that exogenous adenosine significantly enhanced the barrier function in VVEC-Co and, to a lesser extent, in VVEC-Hyp. Our data from a quantitative reverse transcription polymerase chain reaction show that both VVEC-Co and VVEC-Hyp express all four adenosine receptors (A1, A2A, A2B, and A3), with the highest expression level of A1 receptors (A1Rs). However, A1R expression was significantly lower in VVEC-Hyp compared to VVEC-Co. By using an A1R-specific agonist/antagonist and siRNA, we demonstrate that A1Rs are mostly responsible for adenosine-induced enhancement in barrier function. Adenosine-induced barrier integrity enhancement was attenuated by pretreatment of VVEC with pertussis toxin and GSK690693 or LY294002, suggesting the involvement of Gi proteins and the PI3K-Akt pathway. Moreover, we reveal a critical role of actin cytoskeleton in VVEC barrier regulation by using specific inhibitors of actin and microtubule polymerization. Further, we show that adenosine pretreatment blocked the tumor necrosis factor alpha (TNF-α)-induced permeability in VVEC-Co, validating its anti-inflammatory effects.

**Conclusions:**

We demonstrate for the first time that stimulation of A1Rs enhances the barrier function in VVEC by activation of the Gi/PI3K/Akt pathway and remodeling of actin microfilament.

## Introduction

Pathological vascular remodeling plays a pivotal role in the progression of a variety of pulmonary vascular diseases, including pulmonary hypertension [1]. Moreover, many pulmonary vascular diseases are associated with lung exposure to hypoxia and subsequent development of the inflammatory, fibrotic, and angiogenic responses in the vasculature [2], [3], [4], [5]. The *vasa vasorum* is a microcirculatory network that provides oxygen and nutrients to the adventitial and medial compartments of large blood vessels. Although it was originally recognized as the main guardian of vascular integrity, the *vasa vasorum* has recently emerged as an important contributor to the initiation and progression of vascular diseases, through processes of angiogenesis and vasculogenesis [6], [7], [8]. Our recent data in a neonatal model of pulmonary hypertension showed that angiogenic expansion of the *vasa vasorum* network can be observed in the pulmonary arteries of chronically hypoxic calves, and that this process is accompanied by marked adventitial thickening, as well as infiltration and homing of circulating inflammatory cells in the pulmonary artery vascular wall [1], [9], [10].

The vascular endothelium is recognized as an active part of the vasculature due to its secretory and adhesive properties [11], [12]. Moreover, the endothelium is a semi-selective diffusion barrier regulating a variety of functions, including the passage of macromolecules and fluids between the blood and the interstitial fluid. Defects in some physiological functions of the endothelium lead to inflammatory lung disorders, such as pulmonary hypertension and acute lung injury. Elevated expression of intercellular adhesion molecule-1 (ICAM-1) by tumor necrosis factor-alpha (TNF-α) has been described as an important mechanism of leukocyte sequestration in the pulmonary microvasculature in patients with acute lung inflammation [13].

The role of extracellular purine nucleotides (ATP and ADP) and adenosine as important regulators of vascular cell function is well recognized [9], [14], [15], [16], [17], [18]. Adenosine is produced in response to metabolic stress and cell damage, and its levels are elevated in ischemia, hypoxia, inflammation, and trauma [19]. The dominant sources of extracellular adenosine are mainly ATP and ADP that are hydrolyzed by the combined action of ecto-enzymes, CD39/NTPDase-1 and CD73/ecto-5′-nucleotidase [20], [21], [22]. Extracellular adenosine binds to P1, G protein-coupled adenosine receptors (A1, A2A, A2B, and A3) that have been pharmacologically well characterized [23], [24]. Activation of A1 and A3 receptors leads to a decrease in cAMP concentration via inhibition of adenylate cyclase and to a raise in intracellular Ca^2+^ levels by a pathway involving phospholipase C activation [23], [25]. In contrast, stimulation of A2A and A2B receptors leads to activation of adenylate cyclase and generation of cAMP, whose role in the regulation of cell barrier function is well characterized [19], [26]. Adenosine can activate A1, A2A, and A3 receptors with EC_50_ of 0.2–0.7 µM range, whereas the potency of adenosine toward A2B receptors is much lower (EC_50_: 24 µM) [25]. This receptor complexity reflects the multifaceted role played by adenosine in health and disease, including inhibiting of pro-inflammatory responses and preventing excessive tissue damage [24], [27].

Extracellular adenosine has been implicated in the regulation of vascular permeability and inflammation in the vasculature [22], [28], [29], [30]. Studies on CD73(−/−) mice provided evidence that extracellular adenosine reversed hypoxia-induced vascular leakage in different organs, especially in the lung [22]. In addition, studies on adenosine receptor subtype-specific knockout mice demonstrated that this protective effect of adenosine is mediated by A2B receptors [30]. In contrast, activation of A3 receptors with adenosine resulted in increased cutaneous vascular permeability [29]. The key regulatory role of ecto-5′-nucleotidase/CD73 and adenosine in controlling the endothelial barrier function *in vitro* has been supported by studies on transendothelial leukocyte migration [31], [32], [33], [34]. Complementary to these observations, hypoxia-induced vascular leak can be attenuated by an increase in the level of extracellular adenosine due to HIF-1α–dependent repression of adenosine kinase, an enzyme catalyzing adenosine phosphorylation to AMP, and thereby [35]. Since extracellular adenosine is an important physiological regulator of vascular permeability and inflammation, this study was undertaken to further elucidate the adenosine receptor-mediated signaling contributing to VVEC barrier integrity. Our data demonstrate that extracellular adenosine, acting mostly through A1Rs, enhanced the barrier function in VVEC via the mechanisms that involve Gi/PI3K/Akt signaling and actin cytoskeleton remodeling.

## Materials and Methods

### Materials

siPORT Amine transfection reagent was purchased from Ambion (Austin, TX). Adenosine A1 receptor antibody (sc-28995), A1R-specific small interfering ribonucleic acid (siRNA), and horseradish peroxidase-conjugated goat anti-rabbit IgG antibody were procured from Santa Cruz Biotechnology (Santa Cruz, CA). TRIzol was obtained from Invitrogen (Carlsbad, CA). Anti-phospho-Akt (Ser473) and anti-tubulin antibodies were obtained from Cell Signaling Technology (Danvers, MA). An enhanced chemiluminescence detection kit (ECL) was purchased from Amersham (Little Chalfont, UK). Endothelial cell growth supplement was obtained from Millipore (Billerica, MA). The GSK690693 (Akt inhibitor), LY294002 (PI3K inhibitor), adenosine receptors-specific agonists and antagonists were obtained from Tocris Bioscience (Ellisville, MI). Alexa Fluor 488 Phalloidin (Cat # A12379) was purchased from Invitrogen. All other reagents were obtained from Sigma-Aldrich (St. Louis, MO).

### Isolation and culture of VVEC

VVEC were isolated from the pulmonary artery adventitia of normoxic (two-week kept at ambient Denver altitude; P_B_ = 640 mmHg) and chronically hypoxic (two-week exposed to hypobaric hypoxia; P_B_ = 430 mmHg) male Holstein calves as previously described [9]. Standard veterinary care was used following institutional guidelines, and the procedure was approved by the Institutional Animal Care and Use Committee (Department of Physiology, School of Veterinary Medicine, Colorado State Univ., Ft. Collins, CO). Animals were sacrificed by an intravenous overdose of pentobarbital. The protocol was approved by the Institutional Animal Care and Use Committee at Colorado State University. Isolated VVEC have been shown to: express endothelial cell markers, including vWF, eNOS, and PECAM-1; bind the lectin *Licopercsicon esculentum*; and incorporate acetylated low density lipoproteins labeled with 1,1′-dioctadecyl-3,3,3′,3′-tetramethylindo-carbocyanine perchlorate. Cells were grown in high glucose Dulbeccós Modified Eagle-Medium (DMEM), supplemented with 10% fetal bovine serum (FBS), 1% non-essential amino acids, 100 U/ml penicillin, 100 µg/ml streptomycin, 10 mM L-glutamine, and 30 µg/ml endothelial cell growth supplement. VVEC were used in the experiments at passage 2–5.

### Measurement of endothelial monolayer electrical resistance

The barrier properties of VVEC monolayers were characterized using an electrical cell-substrate impedance sensing (ECIS) instrument (Applied Biophysics, Troy, NY) as described previously [15], [36], [37]. Transendothelial electrical resistance (TER) data were normalized to initial voltage. The VVEC were seeded in ECIS arrays until formation of a monolayer for 24–48 h. Before each experiment, VVEC were incubated with serum-free medium for 2 h. After a baseline measurement, cells were treated with various concentrations of adenosine or adenosine receptor-specific agonists, and the TER measurement was monitored for 4–6 h. In other experiments, VVEC were pretreated with the receptor-specific antagonists for 30 min followed by a treatment with adenosine or adenosine receptor-specific agonists.

### Quantitative reverse transcriptase-polymerase chain reaction (qRT-PCR)

The presence of specific mRNA transcripts for A1R was evaluated by qRT-PCR. Cellular mRNA was isolated from 3–4 independent isolations of VVEC from control and high altitude-exposed animals, using an RNease mini kit (Qiagen, Valencia, CA, USA). cDNA was synthesized from 1 µg of RNA, using an iScript cDNA synthesis kit (Bio-Rad, Hercules, CA, USA), according to the manufacturer's specifications. Quantitative RT-PCR was performed to measure A1, A2A, A2B, and A3 mRNA levels, using gene-specific primers: A1 (NM_174497) – sense: ACTTCAACTTCTTCGTGTGGGT, antisense: GCCGACACCTTCTTGTTGAGC; A2A (XM_870153) – sense: CCTCTTCTTCGCCTGCTTTGTCC, antisense: CCCCGTCACCAAGCCGTTGTACC; A2B (NM_001075925) – sense: GATCCCCTTCCAGAACTAGGTG, antisense: TAAAATTTTCACTTTGGGGTCCAG; A3 (NM_001104611) – sense: CTCCATTGTTACTGCTACGTG, antisense: TATCAAGCCCGATCAGTCCC); and ß-actin (NM_001101) – sense: GGCACCACACCTTCTACAA, antisense: AGCCTGGATAGCAACGTAC. The efficiency of the qRT-PCR for four adenosine receptors and a housekeeping gene was 93–98%. Equal amounts of cDNA, equivalent to 5 ng of RNA, were used in each reaction carried out in iTaq Fast SYBR Green Supermix with ROX (Bio-Rad, Hercules, CA, USA) using the ABI 7500 Fast Real-time PCR System (Applied Biosystems, Inc., Foster City, CA, USA). The relative amount of each gene in each sample was estimated by the 2^−Δ/ΔC^_T_ method [38]. The expression of the target genes was normalized to that of the housekeeping gene, ß-actin, in each sample.

### Depletion of endogenous A1R mRNA using siRNA

VVEC were cultured to 60–70% confluence and transfected with siRNA specific to A1R (Ambion) or scrambled siRNA as a control, using siPORT Amine transfection reagent, according to the manufacturer's protocol (Applied Biosystems, Carlsbad, CA). Briefly, cells were serum-starved for 1 h followed by incubation with 20 nM siRNA for 6 h in a low serum medium. Then, fresh medium containing 1% serum was added and 42 h later cells were used in biochemical experiments, ECIS, and/or functional assays. To confirm the A1R depletion, RNA was isolated using TRIzol, and the A1R level was analyzed by RT-PCR. For TER measurement, cells were grown to yield 60–70% confluence in ECIS arrays and transfected with siRNA, as described previously [15].

### Immunoblotting

Protein extracts were separated by SDS-PAGE, transferred to the nitrocellulose membrane, and probed with specific antibodies. Horseradish peroxidase-conjugated goat anti-rabbit IgG antibody was used as the secondary antibody, and immunoreactive proteins were detected using an ECL kit according to the manufacturer's protocol as previously described [9].

### Immunofluorescence microscopy

Immunostaining was performed as described previously [15], [36]. Alexa Fluor 488 Phalloidin, a high-affinity filamentous actin (F-actin) probe, was used to stain actin in VVEC. Images were captured using a confocal microscope (ZEISS) under high magnification (160×, Immersion oil).

### Statistical analysis

All measurements are presented as the mean ± SEM of at least 3 independent experiments. To compare results between groups, a 2-sample Student t test was used. For comparison among groups, 1-way ANOVA was performed. Differences were considered statistically significant at *p*<0.05.

## Results

### Effects of extracellular adenosine on transendothelial electrical resistance (TER) in VVEC

**Figure 1:**
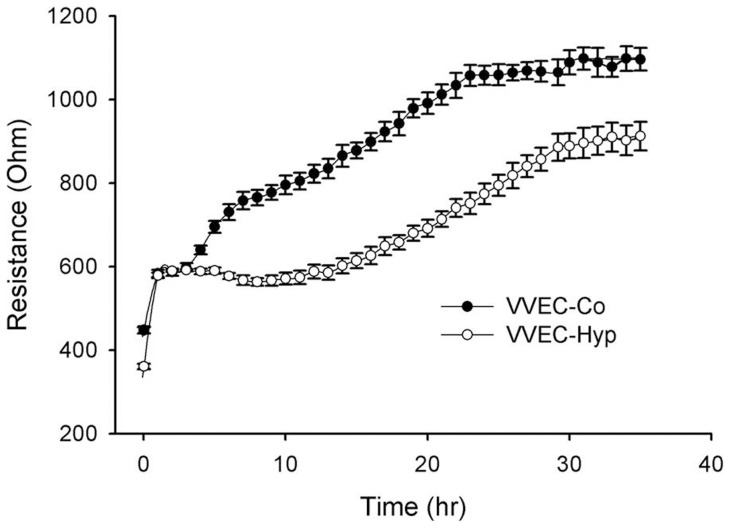
Proliferation of VVEC-Co and VVEC-Hyp in ECIS arrays. Equal numbers of VVEC-Co and VVEC-Hyp (100,000 cells/well) were seeded in ECIS arrays and the TER was measured for 36 h. Results are presented as mean ± SEM and derived from three independent experiments.

**Figure 2:**
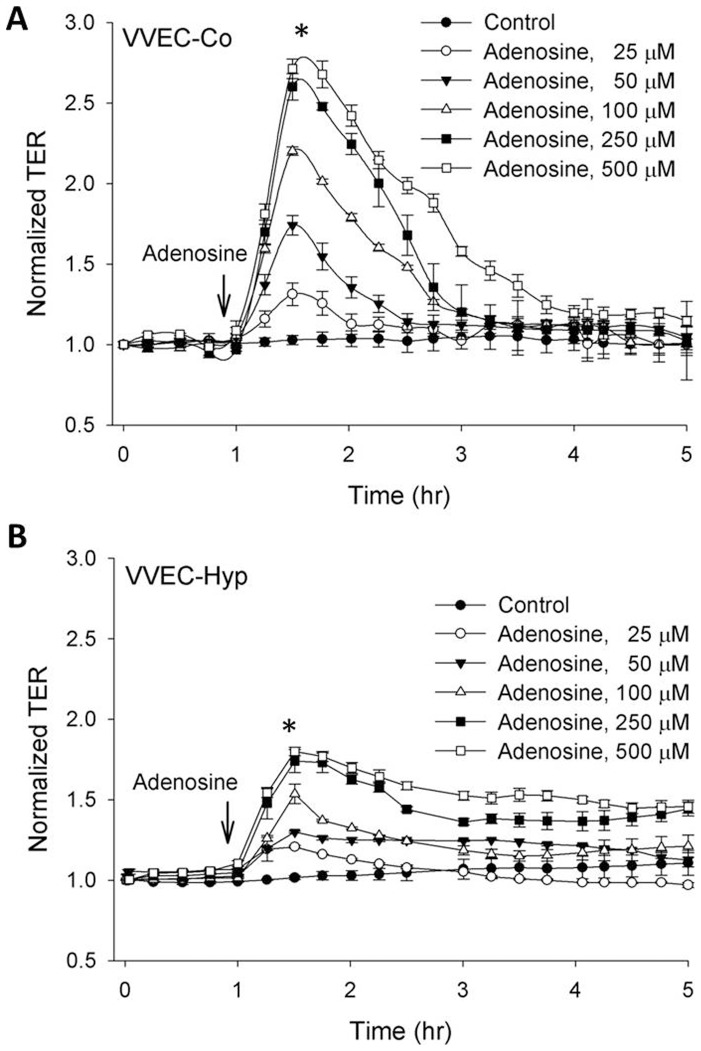
Adenosine enhances the VVEC barrier function. VVEC monolayers in ECIS arrays were incubated in serum free medium for 1 h. Adenosine (50–500 µM) was added to VVEC-Co (**A**) or VVEC-Hyp (**B**) after a steady baseline was established, and the TER measurements continued for 6 h. Data are representative of multiple independent experiments (minimum of three).

Our initial observation demonstrated that VVEC-Co and VVEC-Hyp monolayers exhibit different TER, with lower resistance observed in “hypoxic” cells (Fig. 1). Extracellular adenosine increased the TER of VVEC-Co in a concentration-dependent manner (Fig. 2A), indicating barrier enhancement. A similar but less pronounced effect was observed in VVEC-Hyp (Fig. 2B). One hundred µM adenosine induced a ∼1.7-fold TER increase in VVEC-Hyp (Fig. 2B) versus ∼2.7-fold for VVEC-Co (Fig. 2A). Although the adenosine-induced barrier increase in VVEC-Hyp was relatively lower, the adenosine mediated increase in TER was sustained longer in these cells compared to VVEC-Co, which could be explained by lower initial resistance of VVEC-Hyp compared to VVEC-Co.

### Analysis of expression of adenosine receptors in VVEC by qRT-PCR

**Figure 3:**
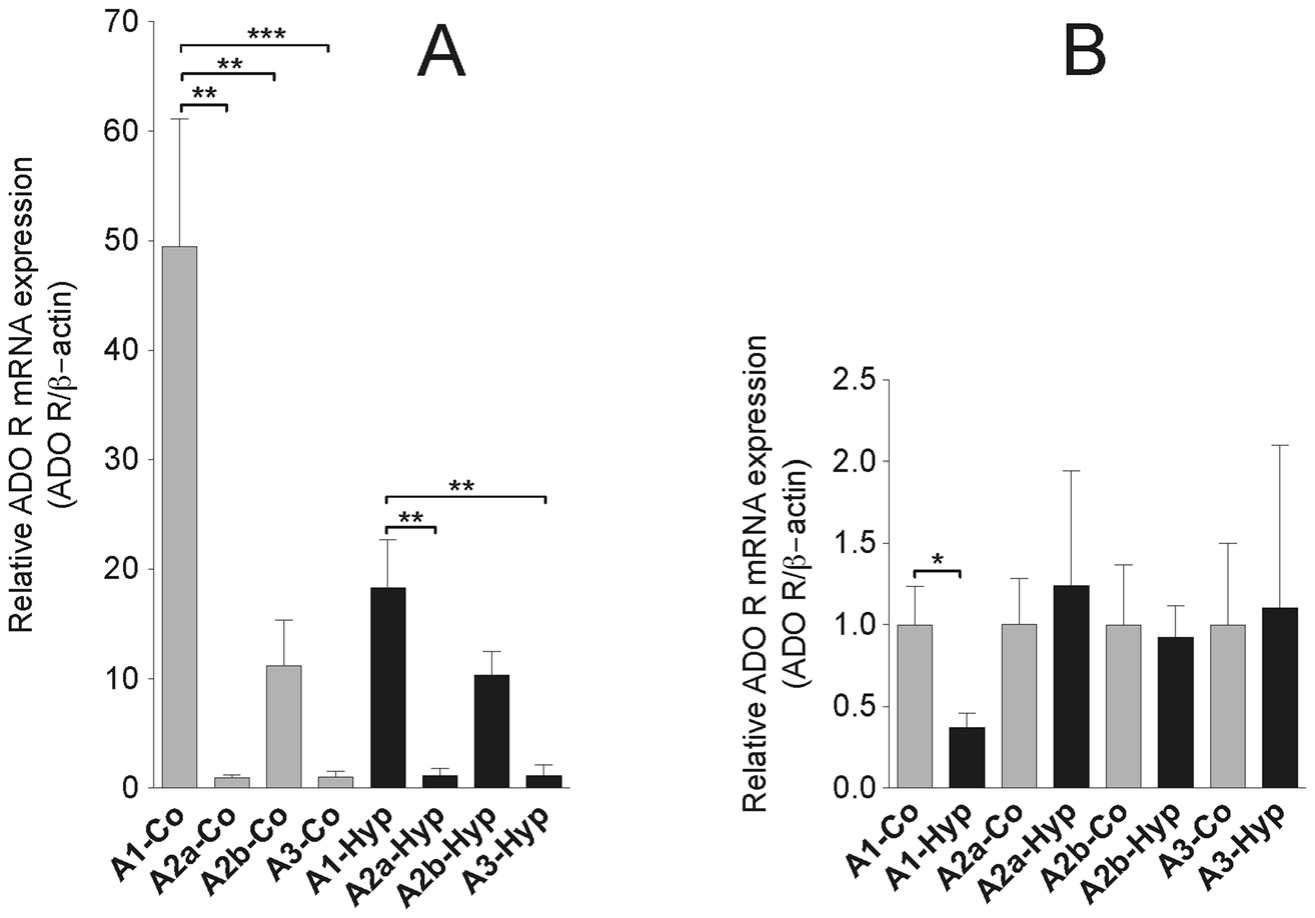
The qRT-PCR analysis of adenosine receptors expression in VVEC. The fold change in mRNA expression of each adenosine receptor relative to internal housekeeping gene (ß-actin) was calculated. (**A**) Adenosine receptor mRNA levels were normalized versus VVEC-Co A3R, whose expression level was arbitrarily established as 1. The results are shown as mean ± SEM from at least three distinct VVEC populations. Data were analyzed by one-way ANOVA followed by Tukey's multiple comparison test for VVEC-Co and VVEC-Hyp separately. (**B**) Adenosine receptor mRNA levels in VVEC-Hyp were normalized to VVEC-Co for each gene. Data were analyzed by Student's t-test. **p<0.01, ***p<0.001.

As adenosine plays an important role in strengthening the EC barrier, we investigated the expression pattern of adenosine receptors in VVEC. Our qRT-PCR data indicate that both VVEC-Co and VVEC-Hyp express all four adenosine receptors, with the highest RNA expression level of A1Rs followed by lower expression levels of A2B, A2A and A3R (Fig. 3A). Moreover, our data indicate that the expression of A1Rs is significantly decreased in VVEC-Hyp compared to VVEC-Co (Figs. 3A and B).

### Identification of adenosine receptors involved in the regulation of VVEC barrier function

**Figure 4:**
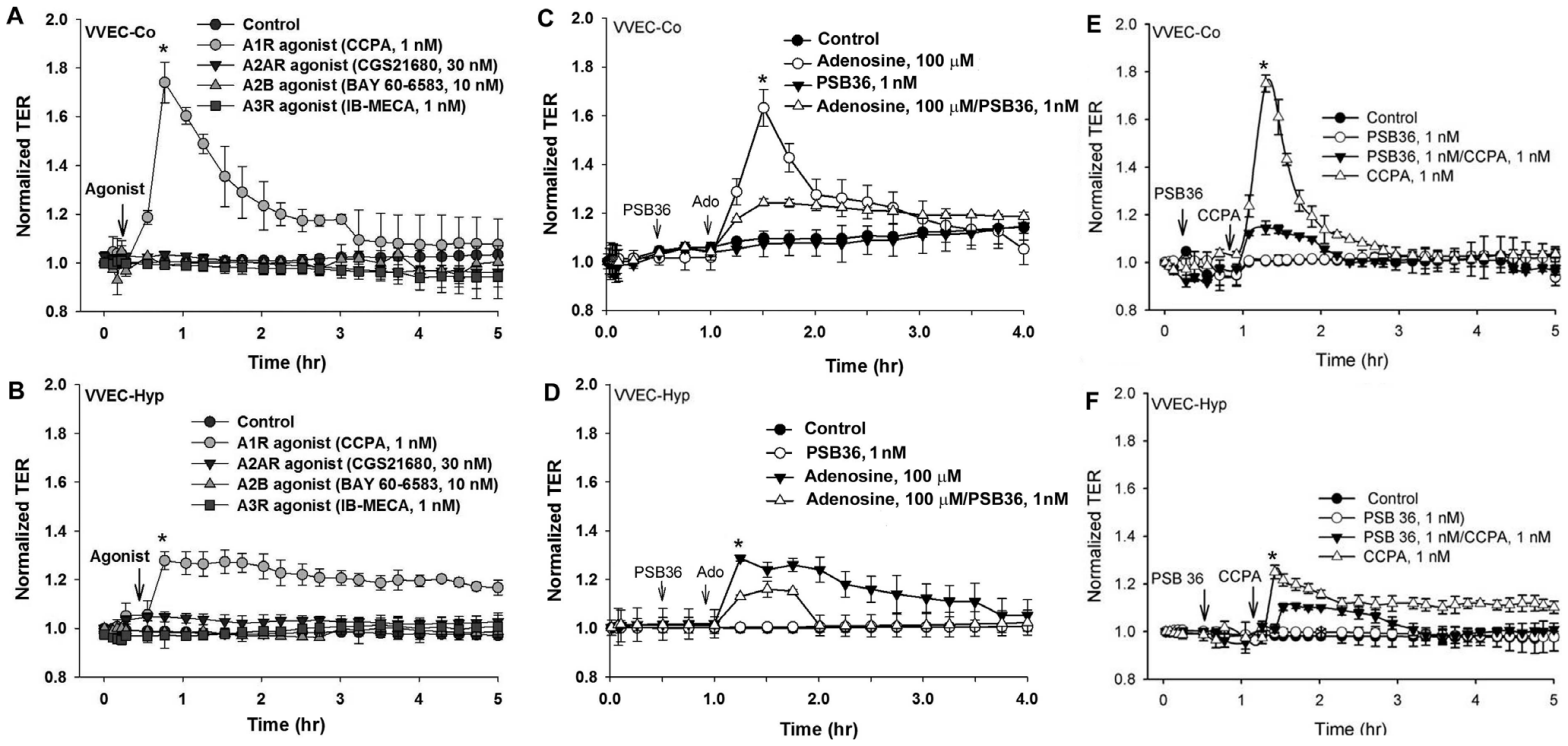
Effects of adenosine receptor agonists on the VVEC barrier function. Activation of A1R improves VVEC barrier function. VVE-Co (**A**) and VVEC-Hyp (**B**) were stimulated with various agonists of adenosine receptors (CCPA, 1 nM; CGS21680, 30 nM; BAY 60-5683 10 nM; IB-MECA, 1 nM) and barrier function was analyzed by TER. VVE-Co (**C**) and VVEC-Hyp (**D**) were stimulated with adenosine (Ado, 100 μM) with and without A1R specific antagonist (PSB 36, 1 nM, 30 min), and barrier function was analyzed by TER. VVE-Co (E) and VVEC-Hyp (F) were stimulated with A1R specific agonist (CCPA, 1 nM) with and without A1R antagonist (PSB 36, 1 nM, 30 min), and barrier function was analyzed by TER.

We used pharmacological and genetic approaches to define the adenosine receptors involved in the regulation of the VVEC barrier function. Minimal effective concentration of each agonist was used. Agonist-treated cells were subjected to TER assay, as described above. Our data indicate that CCPA, an A1R-specific agonist, significantly enhanced the barrier function in both VVEC-Co and VVEC-Hyp (Figs. 4A and B). Intriguingly, specific agonists of A2A, A2B, and A3 adenosine receptors, CGS21680, BAY 60-5683 and IB-MECA, respectively, failed to increase the barrier function (Figs. 4A and B), indicating a pivotal role of A1 receptors in barrier enhancement function.

In order to demonstrate the involvement of A1Rs in adenosine-induced barrier enhancement in VVEC, we used a selective antagonist of A1Rs, PSB-36, as well as specific siRNA. PSB-36 significantly inhibited adenosine-induced TER (Figs. 4C and D). The effect of the A1R agonist, CCPA, on TER was observed in both VVEC-Co and VVEC-Hyp, but was much stronger in the control cells, again suggesting that chronic hypoxia impairs adenosine-induced VVEC barrier regulation.

In VVEC pretreated with PSB-36 the barrier enhancing effect of CCPA was significantly attenuated in both VVEC-Co and VVEC-Hyp (Figs. 4E and F), suggesting that A1Rs play a predominant role in maintaining VVEC barrier function.

**Figure 5:**
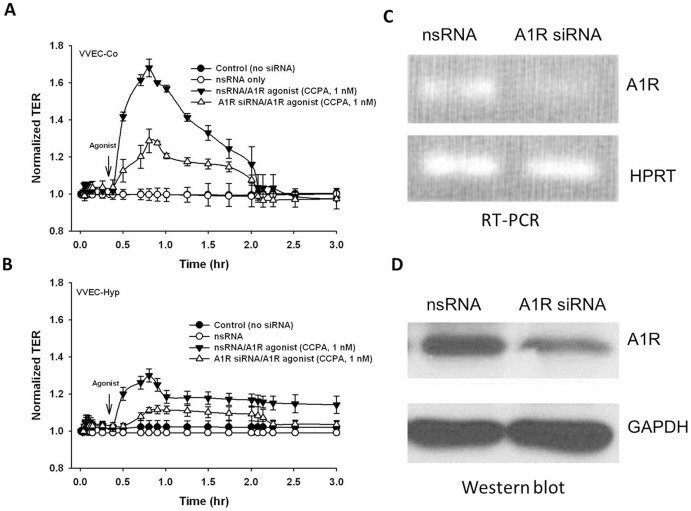
A1R is involved in adenosine-induced VVEC barrier function. Effect of A1R siRNA on CCPA-induced increase in TER in VVEC. (**A, B**) VVEC were incubated with A1R specific siRNA or non-specific siRNA for 48 h and then cells were stimulated with CCPA (1 nM) in TER measurement assay. The depletion of A1R mRNA and protein was confirmed by RT-PCR (**C**) and the Western blot analysis with anti-A1R antibody. (**D**). Results are presented as mean ± SE from three independent experiments.

To further investigate the role of A1R in cell barrier function, VVEC were transfected with a specific and previously validated siRNA to this receptor. Forty-eight hours after transfection, cells were stimulated with A1R-specific agonist CCPA, followed by TER measurement. Our data demonstrate that silencing of A1R attenuated the effects of CCPA in both VVEC-Co and VVEC-Hyp (Figs. 5A and B, respectively), confirming that A1Rs are responsible for the agonist-induced VVEC barrier enhancement. Control scrambled siRNA had no effect on ligand-induced VVEC barrier function. We confirmed the A1R expression inhibition at both RNA and protein levels by RT-PCR (Fig. 5C) and Western blot (Fig. 5D), respectively.

### Role of Gi and Akt signaling in adenosine-induced enhancement of VVEC barrier function

**Figure 6:**
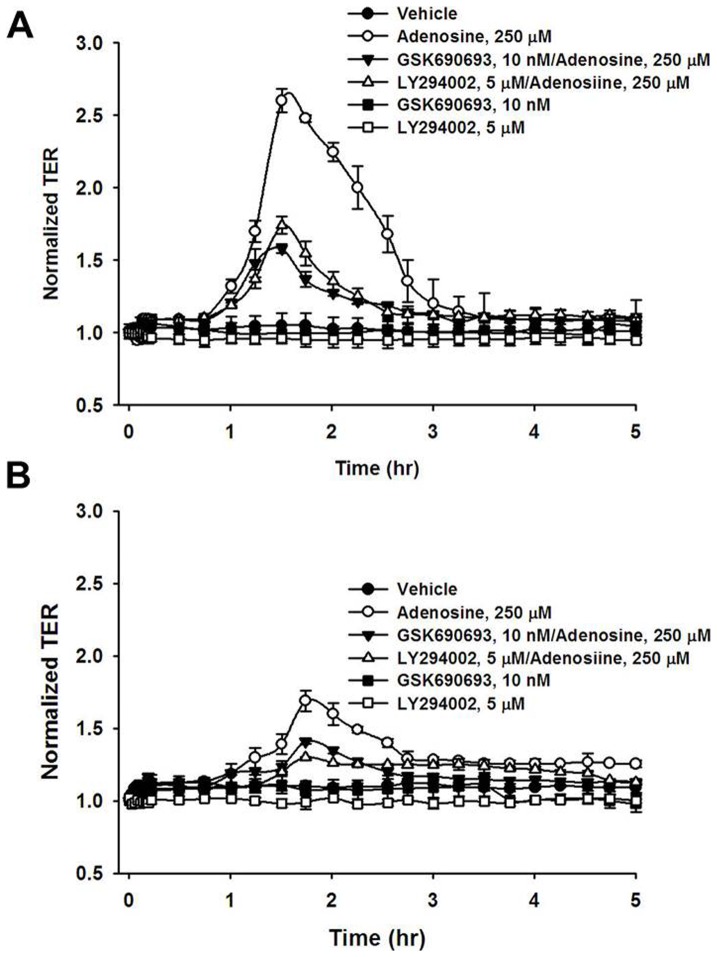
PI3K/Akt pathway mediates adenosine-induced increase in TER in VVEC. VVEC-Co (**A**) and VVEC-Hyp (**B**) were pre-incubated with LY294002 (5 µM; PI3K inhibitor) or GSK690693 (10 nM; Akt inhibitor) for 30 min and then exposed to adenosine. Barrier function was measured by TER assay. Results were obtained from three independent experiments and are presented as mean ± SE. * p<0.05.

**Figure 7:**
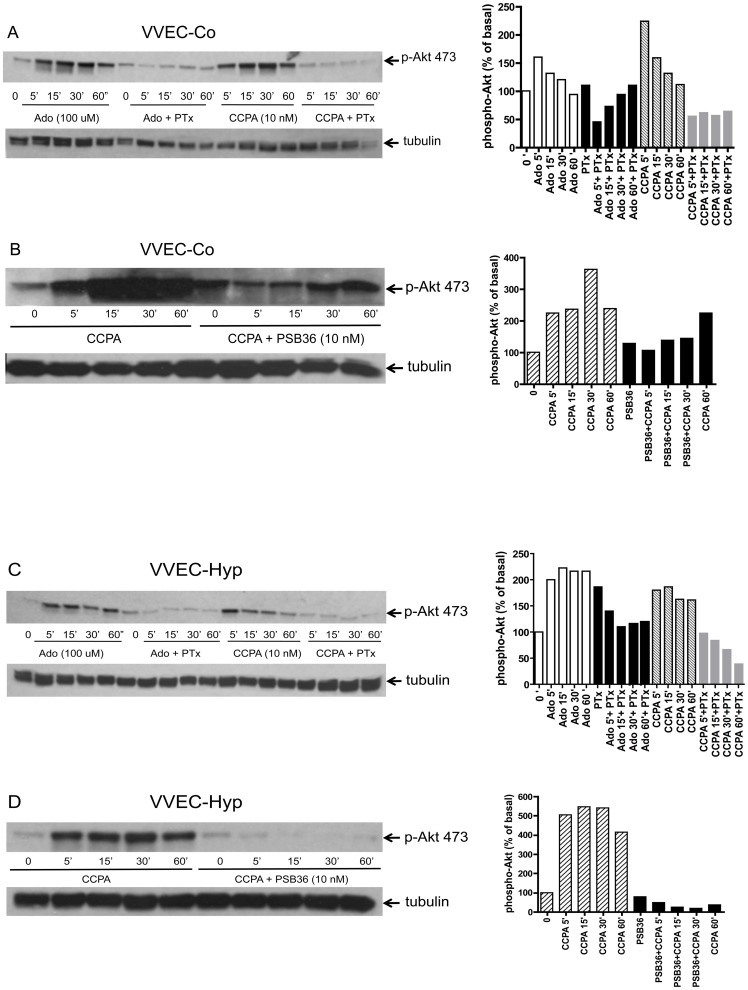
Adenosine-induced AKT phosphorylation in VVEC is mediated via Gαi. To dissect a role of Gi proteins in Akt activation, VVEC-Co (**A**) and VVEC-Hyp (**C**) were pre-treated with PTx (100 ng/ml, 18 h) and stimulated with 100 μM adenosine (Ado) or 10 nM CCPA for the indicated periods of time. To determine the role of adenosine A1R in Akt activation, VVEC-Co (**B**) and VVEC-Hyp (**D**) were pre-treated with 10 nM PSB 36 (30 min), a specific A1R antagonist, followed by stimulation with 100 µM adenosine (Ado) or 10 nM CCPA for the indicated periods of time. Data are representative from at least three independent experiments.

Previous study demonstrated an involvement of the PI3K/Akt pathway in regulating endothelial barrier function in large blood vessels [39]. To test whether this signaling pathway contributes to adenosine-induced enhancement of VVEC barrier function, cells were treated with a specific inhibitor of PI3K (LY294002) or Akt (GSK690693) followed by TER assessment. As shown in Fig. 6, treatment with LY294002 or GSK690693 significantly attenuated adenosine-induced enhancement of barrier function in both VVEC-Co (Fig. 6A) and VVEC-Hyp (Fig. 6B). To further investigate this signaling pathway, we examined Akt phosphorylation by Western blot analysis. Phospho-Akt levels in adenosine- or CCPA-treated VVEC-Co and VVEC-Hyp were significantly increased compared to untreated cells (Figs. 7A and C). The response to CCPA was blunted in the cells pre-treated with PSB-36, indicating that A1Rs are involved in Akt phosphorylation in both VVEC-Co and VVEC-Hyp (Figs. 7B and D). As A1Rs are coupled to Gi proteins, we investigated whether pertussis toxin (PTx), an inhibitor of Gi-dependent signaling, affects Akt phosphorylation in response to adenosine or CCPA stimulation. Pretreatment of VVEC with PTx (100 ng/ml, 18 h) resulted in a substantial decrease of Akt phosphorylation in both adenosine- and CCPA-treated VVE-Co (Fig. 7A) and VVEC-Hyp (Fig. 7C).

### Distinct roles of actin microfilaments and microtubules in the barrier-protective effect of adenosine

**Figure 8:**
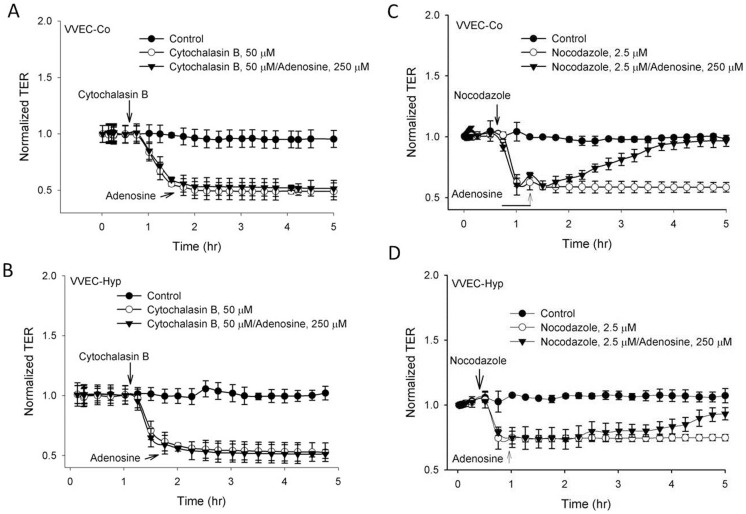
Actin microfilament rearrangement is required for the barrier-protective effect of adenosine. (**A**) VVEC were pretreated with either vehicle or actin depolymerizing agent, cytochalasin B, for 30 min and then stimulated with adenosine (250 µM). Actin depolymerization rapidly decreases the TER and completely prevented the protective effect of adenosine in both VVEC-Co and VVEC-Hyp. (**B**) VVEC were treated with either vehicle or the microtubule-disrupting agent, nocodazole, for 30 min and then stimulated with adenosine (250 µM). Disruption of microtubules also decreases the TER rapidly, but failed to alter the adenosine-induced increases in TER in both VVEC-Co and VVEC-Hyp. Results are presented as mean ± SE and obtained from three independent experiments.

Several studies documented that the endothelial cytoskeleton (primarily actin filaments and microtubules) is a critical determinant of vascular integrity and barrier regulation [40], [41]. To test whether the adenosine-induced barrier protective effect is mediated by stabilization of actin microfilaments or via targeting of the microtubule cytoskeleton, we studied the effect of adenosine on VVEC hyperpermeability after actin microfilament disruption by cytochalasin B or microtubule disassembly by nocodazole. Cytochalasin B treatment of both VVEC-Co and VVEC-Hyp resulted in a rapid and dramatic decrease in TER. Treatment with adenosine at the point when the decrease in TER reached its lowest point had no protective effect on cytochalasin B-induced VVEC hyperpermeability (Figs. 8A and B), suggesting that actin microfilament integrity is required for the barrier-protective effect of adenosine. Pretreatment of VVEC with nocodazole, a microtubule depolymerizing/disrupting agent, also resulted in a rapid and dramatic decrease in TER. But in contrast to the effects of cytochalasin B, nocodazole-induced VVEC permeability was completely restored by adenosine (Figs. 8C and D), suggesting that microtubule disruption is not an essential component in adenosine-induced enhancement of VVEC barrier function.

### Analysis of extracellular adenosine-induced actin cytoskeleton rearrangements

**Figure 9:**
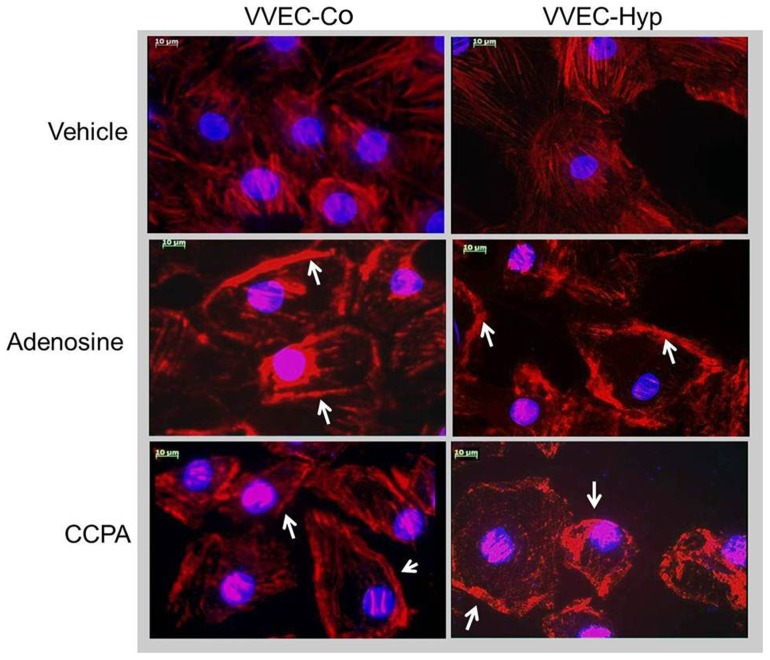
Effect of adenosine on actin cytoskeleton in VVEC. VVEC-Co and VVEC-Hyp were incubated with or without 250 μM adenosine for 30 min. Actin staining was assessed with a high-affinity F-actin probe, Alexa Fluor 488 Phalloidin. Immunofluorescence images were captured using a confocal microscope under 160× magnification (Immersion oil). Representative cells were selected from groups of VVEC-Co and VVEC-Hyp.

To study the effect of adenosine on the actin cytoskeletal arrangement in VVEC, we performed an immunocytochemical analysis of actin filaments. The cell monolayers were treated with either vehicle or adenosine for 30 min, and Alexa Fluor 488 Phalloidin was used for F-actin staining. Our data indicate that adenosine treatment significantly increased the polymerized cortical actin formation in the cell-cell junctions of VVEC-Co compared to vehicle-treated cells (Fig. 9). Similar, but weaker adenosine-induced cortical actin formation was observed in VVEC-Hyp. These data further demonstrate that actin reorganization may play an important role in adenosine-induced barrier enhancement in VVEC.

### Effect of TNF-α on the VVEC barrier function

**Figure 10:**
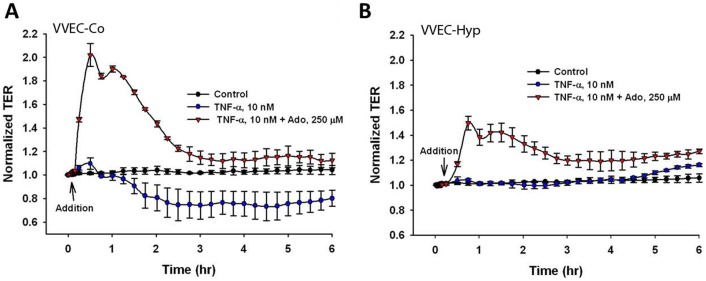
Effect of TNF-α on the VVEC barrier function. VVEC monolayers (VVEC-Co and VVEC-Hyp) were treated with TNF-α (50 nM) and the TER was measured in ECIS arrays. Results are presented as mean ± SE and obtained from three independent experiments.

TNF-α, one of the most potent pro-inflammatory factors, regulates vascular endothelial cell permeability through stress fiber formation and interruption of cellular junctions [42], [43], [44]. To analyze the effect of TNF-α on VVEC barrier function, TER was monitored in cells incubated with TNF-α. Our data indicate that TNF-α decreased TER in VVEC-Co, which translates to increased cell permeability, and this effect persisted for several hours (Fig. 10A). In contrast, TNF-α failed to increase the permeability of the VVEC-Hyp, possibly due to impaired barrier function of VVEC-Hyp under basal conditions (as indicated in Fig. 1). Simultaneous addition of TNF-α and adenosine resulted in a dramatic increase in TER, suggesting that the barrier-protective effect of adenosine may overcome TNF-α-mediated cell permeability (Fig. 10).

## Discussion

Exposure to hypoxia induces a vascular leakage leading to pulmonary edema, vascular inflammation, and angiogenesis. In our previous study we used a neonatal model of hypoxia-induced pulmonary hypertension and we demonstrated marked vascularization of the *vasa vasorum* network that was accompanied by infiltration and homing of circulating progenitor and inflammatory cells in the pulmonary artery vascular wall [45]. Although endothelial dysfunction and permeability changes have been intensively investigated in pulmonary artery endothelial cells, the mechanisms that control the pulmonary *vasa vasorum* permeability remain largely unexplored. As extracellular adenosine is an important regulator of vascular inflammation and permeability, in this study we investigated the role of adenosine signaling in VVEC barrier function. First, we demonstrated differential expression of adenosine receptors in VVEC originating from animals kept under normoxic and hypoxic conditions. Second, we presented adenosine-induced VVEC barrier enhancement. Third, using highly selective agonists and antagonists, and receptor-specific siRNA, we established the pivotal role of A1R in VVEC barrier enhancement. Fourth, we showed that A1R acting via Gi-mediated Akt activation was involved in adenosine-induced VVEC barrier enhancement. Fifth, we demonstrated that TNF-α was unable to further impair barrier function in VVEC-Hyp, (contrary to VVEC-Co), suggesting that exposure of VVEC to chronic hypoxia impairs these cells' permeability. Finally, we showed a significant attenuation of TNF-α-induced VVEC permeability upon adenosine treatment, indicative of the barrier-protective effect of adenosine.

The data on the cell growth/proliferation of both control and hypoxic VVEC indicate significantly reduced TER in VVEC-Hyp compared to VVEC-Co from the beginning of the cell spreading until the formation of monolayers. In addition, the monolayers formed from the VVEC-Hyp attained confluence at lower TER values in agreement with our previous observation that these cells are leaky [40] and therefore more fragile to the inflammatory agents. These data are also consistent with the observations from the porcine model of pulmonary hypertension, demonstrating that cells from hypertensive animals showed a higher basal permeability than normal cells [46].

Extracellular nucleotides are well recognized as critical regulators of vascular cell phenotype and function [14], [15], [17], [18], however, little is known about their role in the regulation of endothelial barrier function. Previous study has shown that extracellular ATP exerts a barrier-enhancing effect in human pulmonary artery endothelial cells [47]. Extracellular adenosine, a product of ATP hydrolysis, has long been known to play a protective role against vascular leak under conditions associated with hypoxia and inflammation. Studies from CD73(−/−) mice provided evidence that extracellular adenosine reverses hypoxia-induced vascular leakage in different organs, especially in the lung [22]. In agreement with previous findings, this study demonstrates potent concentration-dependent effects of extracellular adenosine on the VVEC TER. The response was observed in VVEC isolated from both control and chronically hypoxic animals, but the cells from control animals exhibited higher amplitude and shorter duration of the response, whereas the cells from hypoxic animals exhibited lower amplitude and longer duration of the response, indicating that hypoxia-induced alterations of cellular mechanisms involved VVEC barrier function.

Previous studies demonstrated a protective role of A2B adenosine receptors in hypoxia-induced vascular leak in adenosine receptor-knockout mice [19], [30]. Consistent with this observation, a recent report indicated that permeability of pulmonary artery endothelial cells is regulated by A2A and A2B adenosine receptors and an adenosine transporter, pointing out an importance of both extracellular and intracellular adenosine [48]. Results from another study showed that activation of A3R with adenosine and inosine increased cutaneous vascular permeability [19]. Our quantitative RT-PCR data indicate that all four adenosine receptors are expressed in VVEC, with the highest mRNA level observed for A1R, and the lowest for A3. Using pharmacological and genetic approaches, we concluded that adenosine's effect on VVEC permeability is mediated mostly by A1R, while A2AR, A2BR and A3R are not likely to be involved. Importantly, a decrease in expression of A1R in VVEC from hypoxic animals correlates with a lower TER in VVEC-Hyp compared to VVEC-Co. The evidence of A1R involvement in barrier protection is also consistent with an anti-inflammatory role of A1R in several tissues, and may explain both anti-inflammatory and barrier-protective functions of A1R in *vasa vasorum* endothelium. Accordingly, spinal cords and macrophages from A1R(−/−) mice expressed higher levels of pro-inflammatory genes in a model of experimental allergic encephalomyelitis [49], suggesting again that anti-inflammatory signals are mediated by A1R. As previously demonstrated in cell and animal models, A1R was also involved in protective effects against ischemia/reperfusion cell injury [50], [51]. Recent studies reported that A1R in lung microvascular endothelial cells participates in microvascular permeability and leukocyte transmigration [52], and in anti-inflammatory preconditioning [53]. Data from animal models also indicate the involvement of A1R in attenuation of endotoxin-induced lung injury, pulmonary edema, and alveolar destruction. Activation of adenosine A1 and A2 receptors have also been shown to reduce endotoxin-induced cellular energy depletion and oedema formation in the lung [54]. However, our findings are different from the results in human lung microvascular endothelial cells, which demonstrated a role of A2AR in adenosine-induced barrier enhancement [36], [55]. More data are needed to establish whether the concentrations of agonists for the A2A, A2B, and A3R used in our experimental system may indeed trigger the activation of bovine adenosine receptors.

**Figure 11:**
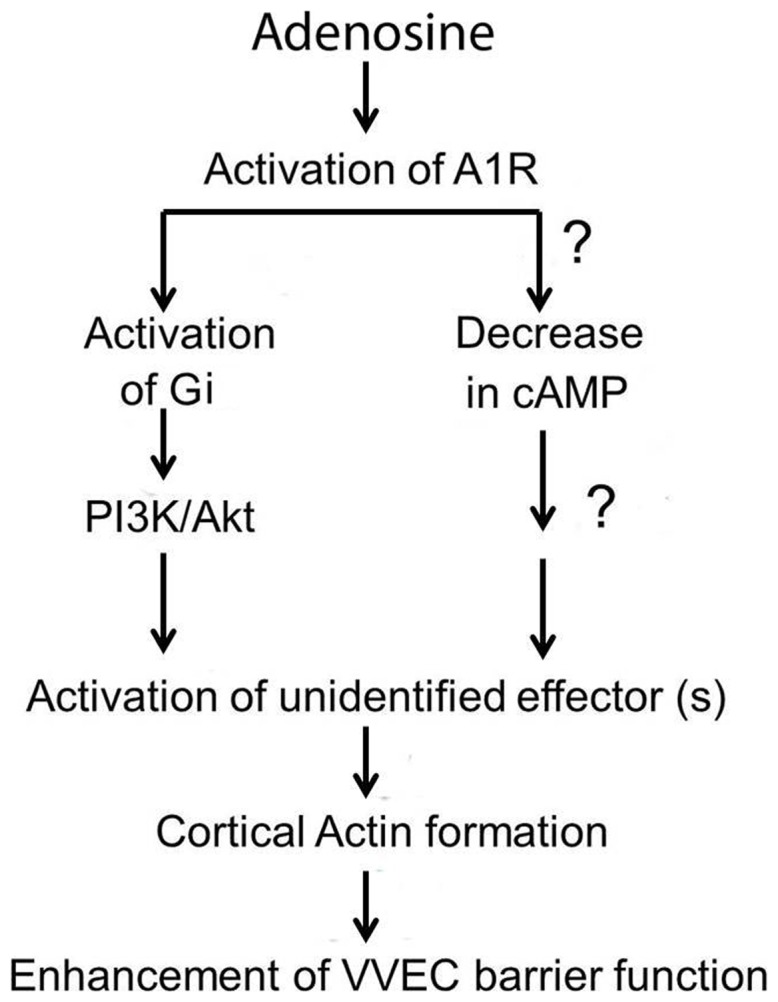
Schematic representation of the proposed signaling pathway of adenosine-induced enhancement of barrier function in VVEC.

The mechanisms that modulate endothelial barrier function were investigated in many studies. In general, the mechanisms that regulate endothelial barrier enhancement are less understood than the mechanisms involved in endothelial barrier disruption. Several ligands, such as sphingosine-1-phosphatase (S1P1), Atrial natriuretic peptide (ANP) and Hapatocyte growth factor (HGF), are reported to enhance or improve endothelial barrier function [56], [57], [58]. It was established in various endothelial cell models that this response involves the activation of cAMP/PKA, cAMP/exchange protein activated by cAMP (EPAC)/Rab, and/or GSK-3β/cathenin, leading to junctional integrity and attenuation of RhoA/ROCK-dependent stress fiber formation [59], [60], [61], [62]. Strikingly, greater paracellular permeability of VVEC-Hyp compared to VVEC-Co does not correlate with the ability of VVEC to produce cAMP in response to forskolin [63]. Our preliminary data also suggest that EPAC is not involved in adenosine-induced VVEC barrier enhancement (data not shown). In this study, we provide clear evidence of the involvement of the Gi/PI3K/Akt pathway in A1R-mediated VVEC barrier enhancement (Fig. 11). Consistent with A1R coupling to Gi, the effects of adenosine and CCPA were attenuated by pretreatment with PTx, which prevents Gi-A1R interaction. Since VVEC express PI3Kβ isoform, which is regulated by Gi-derived βγ subunits [9], a contribution of PI3Kβ in A1R-mediated VVEC barrier function cannot be excluded. We propose that the Gi/PIK3β/Akt pathway represents a novel mode of cytoskeleton remodeling and barrier regulation in VVEC. These findings can be relevant to better understanding of fundamental, tissue-specific mechanisms of microvascular permeability and suggest new therapeutic approaches for endothelial barrier regulation.

Cortical actin formation is associated with endothelial barrier enhancement [64]. We demonstrated that adenosine and CCPA indeed induce cortical actin formation in VVEC. Moreover, we showed that Akt is involved in adenosine-induced barrier regulation. Akt has already been linked to cytoskeletal remodeling in human lung endothelial cells. It was documented that Akt mediates oxidized phospholipid-induced endothelial barrier enhancement by transactivation of the S1P1 receptor, which was followed by Rac1 activation and cortical actin polymerization [56]. Among other proteins, the actin interacting protein Girdin was identified as a novel Akt target contributing to actin cytoskeleton remodeling during cell migration and lamellipodia formation [65]. Intriguingly, a recent study demonstrated that AMPKα1 is co-localized with the adherens junction protein N-cadherin and contributes to endothelial barrier enhancement [66]. An involvement of PI3K/Akt and possibly AMPK signaling in A1R-mediated actin cytoskeleton remodeling and barrier regulation in VVEC remains to be investigated.

TNF-α, one of the most potent pro-inflammatory factors, regulates vascular endothelial cell permeability through stress fiber formation and interruption of cellular junctions [42], [43], [44]. TNF-α expression level and activity can be up-regulated under hypoxia, inflammation, and pulmonary hypertension [67], [68], [69], [70]. It has been shown that among several cell types, macrophages and perivascular adipocytes are potent sources of TNF-α [69], [71]. As the presence of macrophages was observed in pulmonary artery adventitia of chronically hypoxic animals [10], it can be expected that TNF-α, may have a paracrine effect on adventitial *vasa vasorum* in the pulmonary artery wall. The data from this study also show that TNF-α decrease the TER in VVEC-Co, and this effect of TNF-α was blunted by adenosine. Interestingly, TNF-α failed to decrease TER in VVEC isolated from hypoxic animals. This suggests a possibility of persistent phenotypical changes in VVEC in response to chronic hypoxia that could involve TNF-α and adenosine receptors, as well as components of intracellular signaling pathways. A possibility of hypoxia-induced changes in VVEC phenotype is supported by our recently published observation showing the inability of A2A receptor agonists to restore barrier function in VVEC isolated from hypoxic, but not control, animals [63].

In conclusion, in this study we showed for the first time that the adenosine-induced signaling pathway mediated by Gi-coupled A1Rs and PI3K/Akt leads to actin cytoskeleton remodeling and to barrier enhancement in VVEC. In a view of pathologic consequence of hypoxia-induced *vasa vasorum* neovascularization and its function as a conduit for circulating inflammatory cells to the vascular wall, our data indicate that down-regulation of A1R in chronic hypoxia may represent a pathological mechanism of dysregulation of *vasa vasorum* barrier function. This may lead to pulmonary vascular remodeling and inflammation, such as that observed in hypoxic pulmonary hypertension. We propose that A1Rs can be recognized as a vascular bed-specific and novel therapeutic target to regulate *vasa vasorum* barrier function and pathologic vascular remodeling in chronic hypoxia.
