# Correction: Adenosine A1 Receptors Promote *Vasa Vasorum* Endothelial Cell Barrier Integrity via G_i_ and Akt-Dependent Actin Cytoskeleton Remodeling

**DOI:** 10.1371/annotation/42ab28e5-36fe-4a5f-b6aa-5cfec1bf2e63

**Published:** 2013-05-01

**Authors:** Nagavedi Siddaramappa Umapathy, Elzbieta Kaczmarek, Nooreen Fatteh, Nana Burns, Rudolf Lucas, Kurt R. Stenmark, Alexander D. Verin, Evgenia V. Gerasimovskaya

The name of the first author was presented incorrectly in the citation. The correct version of the citation is:

Umapathy NS, Kaczmarek E, Fatteh N, Burns N, Lucas R, et al. (2013) Adenosine A1 Receptors Promote Vasa Vasorum Endothelial Cell Barrier Integrity via Gi and Akt-Dependent Actin Cytoskeleton Remodeling. PLoS ONE 8(4): e59733. doi:10.1371/journal.pone.0059733

There was an error in the Funding statement. The correction Funding statement should be:

This study was supported, in part, by grants, NSU (Biomedical Research Grant, American Lung Association, Southeast), ADV (NHLBI # HL 083327), EVG (NHLBI # 086783), RL (NHLBI # HL094609), and EK (NHLBI # HL106227). The funders had no role in study design, data collection and analysis, decision to publish, or preparation of the manuscript. 

